# [^111^In-DOTA]Somatostatin-14 analogs as potential pansomatostatin-like radiotracers - first results of a preclinical study

**DOI:** 10.1186/2191-219X-2-25

**Published:** 2012-06-09

**Authors:** Aikaterini Tatsi, Theodosia Maina, Renzo Cescato, Beatrice Waser, Eric P Krenning, Marion de Jong, Paul Cordopatis, Jean Claude Reubi, Berthold A Nock

**Affiliations:** 1 Molecular Radiopharmacy, Institute of Radioisotopes - Radiodiagnostic Products, National Center for Scientific Research “Demokritos”, 153 10 Ag. Paraskevi Attikis, Athens, GR-153 10, Greece; 2 Department of Pharmacy, University of Patras, Patras, GR-26500, Greece; 3 Division of Cell Biology and Experimental Cancer Research, Institute of Pathology, University of Berne, Berne, CH-3010, Switzerland; 4 Department of Nuclear Medicine, Erasmus MC, Rotterdam, 3015 GD, The Netherlands

**Keywords:** Radiolabeled pansomatostatins, ^111^In-radiotracer, sst_1-5_-targeted tumor imaging

## Abstract

**Background:**

In this study, we report on the synthesis, radiolabeling, and biological evaluation of two new somatostatin-14 (SS14) analogs, modified with the universal chelator DOTA. We were interested to investigate if and to what extent such radiotracer prototypes may be useful for targeting sst_1-5_-expressing tumors in man but, most importantly, to outline potential drawbacks and benefits associated with their use.

**Methods:**

AT1S and AT2S (DOTA-Ala^1^-Gly^2^-c[Cys^3^-Lys^4^-Asn^5^-Phe^6^-Phe^7^-Trp^8^/DTrp^8^-Lys^9^-Thr^10^-Phe^11^-Thr^12^-Ser^13^-Cys^14^-OH], respectively) were synthesized on the solid support and labeled with ^111^In. The sst_1-5_ affinity profile of AT1S/AT2S was determined by receptor autoradiography using [Leu^8^,dTrp^22^,^125^I-Tyr^25^]SS28 as radioligand. The ability of AT2S to stimulate sst_2_ or sst_3_ internalization was qualitatively analyzed by an immunofluorescence-based internalization assay using hsst_2_- or hsst_3_-expressing HEK293 cells. Furthermore, the internalization of the radioligands [^111^In]AT1S and [^111^In]AT2S was studied at 37 °C in AR4-2J cells endogenously expressing sst_2_. The *in vivo* stability of [^111^In]AT1S and [^111^In]AT2S was tested by high-performance liquid chromatography analysis of mouse blood collected 5 min after radioligand injection, and biodistribution was studied in normal mice. Selectively for [^111^In]AT2S, biodistribution was further studied in SCID mice bearing AR4-2J, HEK293-hsst_2A_^+^, -hsst_3_^+^ or -hsst_5_^+^ tumors.

**Results:**

The new SS14-derived analogs were obtained by solid phase peptide synthesis and were easily labeled with ^111^In. Both SS14 conjugates, AT1S, and its DTrp^8^ counterpart, AT2S, showed a pansomatostatin affinity profile with the respective hsst_1-5_ IC_50_ values in the lower nanomolar range. In addition, AT2S behaved as an agonist for sst_2_ and sst_3_ since it stimulated receptor internalization. The ^111^In radioligands effectively and specifically internalized into rsst_2A_-expressing AR4-2J cells with [^111^In]AT2S internalizing faster than [^111^In]AT1S. *Ex vivo* mouse blood analysis revealed a rapid degradation of both radiopeptides in the bloodstream with the DTrp^8^ analog showing higher stability. Biodistribution results in healthy mice were consistent with these findings with only [^111^In]AT2S showing specific uptake in the sst_2_-rich pancreas. Biodistribution of [^111^In]AT2S in tumor-bearing mice revealed receptor-mediated uptake in the AR4-2J (1.82 ± 0.36 %ID/g - block 0.21 ± 0.17 %ID/g at 4 h post injection (pi)), the HEK293-hsst_2A_^+^ (1.49 ± 0.2 %ID/g - block 0.27 ± 0.20 %ID/g at 4 h pi), the HEK293-hsst_3_^+^ (1.24 ± 0.27 %ID/g - block 0.32 ± 0.06 %ID/g at 4 h pi), and the HEK293-hsst_5_^+^ tumors (0.41 ± 0.12 %ID/g - block 0.22 ± 0.006 %ID/g at 4 h pi). Radioactivity washed out from blood and background tissues via the kidneys.

**Conclusions:**

This study has revealed that the native SS14 structure can indeed serve as a motif for the development of promising pansomatostatin-like radiotracers. Further peptide stabilization is required to increase *in vivo* stability and, consequently, to enhance *in vivo* delivery and tumor targeting.

## Background

Somatostatin-14 (SS14) is a native peptide hormone exerting a variety of physiological actions in the brain and in peripheral tissues after binding to high affinity receptors on the cell membrane of target cells [1-3]. Somatostatin receptors comprise five subtypes (sst_1-5_) and are also found in many human tumors where they are expressed alone or in various combinations [4-7]. Accordingly, they can serve as molecular targets for therapeutic interventions with somatostatin analogs. Native SS14 binds with nanomolar affinity to all five human receptor subtypes, hsst_1-5_, but its use for drug development is prevented by its poor *in vivo* stability [8]. This problem has been competently addressed by the advent of synthetic somatostatin analogs tailored to withstand enzymatic attack *in vivo*, such as octreotide (SMS 201–995, Sandostatin) [9] or Lanreotide (BIM 23014, Somatuline) [10]. Despite their higher potency and longer duration of action, these cyclic-octapeptide analogs have inadvertently become sst_2_-preferring and have lost most of somatostatin’s affinity for the other subtypes. Yet, they have been used with success in the treatment of acromegaly and sst_2_-expressing tumors [11,12].

In a rather recent approach, metabolically stabilized somatostatin analogs have been functionalized with metal chelators to accommodate radiometals useful for diagnostic imaging and radionuclide therapy [13]. ^111^In-DTPA]octreotide (OctreoScan®) is the first approved sst_2_-avid peptide radiopharmaceutical. When administered in patients, it localizes in primary and metastatic sst_2_^+^-lesions, which can be efficiently visualized with the aid of an external imaging device [14]. Following OctreoScan®, several other sst_2_-seeking radiopeptides, suitable for SPECT (^99m^Tc-, ^111^In-, ^67^ Ga-labeled), PET (^68^ Ga-, ^64^Cu-labeled), or radionuclide therapy (^90^Y- or ^177^Lu-labeled) have been evaluated in animal models and in patients with neuroendocrine tumors (NETs) [15-19].

In all above instances, the sst_2_ subtype prevails in incidence and density of expression allowing the successful application of sst_2_-preferring radioligands. However, it should be stressed that, despite the predominance of sst_2_ expression in many human tumors, co-expression of sst_2_ with other sst_1-5_ subtypes is frequent enough. Thus, sst_2_ and sst_5_ are expressed often together in GH-secreting pituitary adenomas, and various combinations of ssts, such as sst_2_ and sst_1_, are expressed in gastroenteropancreatic (GEP)-NETs.

Moreover, a number of human tumors devoid of sst_2_ may instead express one or more of the other sst_1-5_[6,7,15,20-22]. For example, ductal pancreatic carcinomas or primary hormone-sensitive prostate cancers are reported to often express sst_1_[23-26]. Hence, the use of pansomatostatin-like agents will broaden the clinical indications and will increase the diagnostic/therapeutic efficacy of currently available sst_2_-preferring (radio)peptides.

SOM230 and KE108 are two multi-somatostatin receptor ligands that have been developed to improve somatostatin analog-based therapy. SOM230 has high affinity for sst_1-3_ and sst_5_[27], while KE108 has high affinity for all five sst_1-5_[28]. However, the absence of sst_2_ internalization may turn out to be a serious disadvantage of SOM230- or KE108-based radioligands compromising their accumulation in target cells, in the most frequent cases where sst_2_ expression prevails [29-32]. On the other hand, well sst_2_-internalizing and multi-sst_2_-/sst_3_-/sst_5_-binding analogs, such as DOTA-NOC [33], will miss sst_1_-expressing tumors. Thus, a pansomatostatin affinity profile and the preservation of important pharmacological traits, especially sst_2_ internalization, seem to represent an advantageous combination for enhancing the efficacy of sst_1-5_-targeting radioligands.

In this respect, the parent SS14 motif has drawn our attention despite its suboptimal metabolic stability [8]. In fact, not much is reported on the *in vivo* performance of radiopeptides based on SS14. In a previous study, ^111^In-[DTPA,DAla^1^,DTrp^8^,Tyr^11^SS14 showed specific and comparable to OctreoScan® accumulation in physiological sst_2_-rich tissues in mice [34], implying that SS14-based radioligands may indeed possess sufficient *in vivo* stability to successfully reach their target while still able to internalize via the sst_2_.

In this study, we have coupled the universal chelator DOTA to Ala^1^ of SS14 (AT1S). In this way, labeling options beyond ^111^In are feasible while N-terminal capping of SS14 is also achieved, a method known to prolong the biological half-life of peptides. In the second analog, AT2S, Trp^8^ was replaced by dTrp^8^ to further enhance stability [35]. This modification is also reported to improve sst_2_ affinity by favoring the β-turn structure for several cyclic somatostatin analogs [36]. Detailed biological characterization of the AT1S prototype and its DTrp^8^ analog, AT2S, is presented herein encompassing *in vitro* binding affinity and functional assays in sst_1-5_-expressing cells, metabolic studies, and biodistribution of ^111^In-radioligands in mice bearing sst_2_^+^, sst_3_^+^, and sst_5_^+^ tumors. This comprehensive study will provide the basis for structural interventions on the AT1S motif towards improved pansomatostatin-like radiopeptides with advantageous key pharmacological features, such as a preserved sst_2_-internalization capacity.

## Methods

### Chemistry

#### *General*

All chemicals were reagent grade and used without further purification. The protected chelator 2-(4,7,10-tris(2-tert-butoxy-2-oxoethyl)-1,4,7,10-tetraazacyclo-dodecan-1-yl)acetic acid (DOTA-tris(^t^Bu)ester) was supplied by CheMatech (Dijon, France). The l-amino acid precursors, Fmoc-Ala-OH, Fmoc-Gly-OH, Fmoc-Cys(Trt)-OH, Fmoc-Lys(Boc)-OH, Fmoc-Asn(Trt)-OH, Fmoc-Phe-OH, Fmoc-Trp(Boc)-OH, Fmoc-Thr(^t^Bu)-OH, Fmoc-Ser(^t^Bu)-OH, and the d-amino acid precursor, Fmoc-DTrp(Boc)-OH and H-L-Cys(Trt)-2-Chlorotrityl resin (substitution 0.55 mmol/g) that was used in solid-phase peptide synthesis (SPPS), were purchased from CBL (Patras, Greece). [Tyr^3^octreotate (Tate, H-DPhe-c[Cys-Tyr-DTrp-Lys-Thr-Cys]-Thr-OH) and Demopan 2 (DP2, N_4_-Tyr-c[DDab-Arg-Phe-Phe-DTrp-Lys-Thr-Phe]) used for *in vitro* and/or *in vivo* receptor blockade were synthesized as previously described [31,37]. Final purifications were conducted on a semi-preparative high-performance liquid chromatography (HPLC) system Mod.10 ÄKTA from Amersham Biosciences (Piscataway, NJ, USA) on a Supelcosil C18 (5 μm, 8 × 250 mm) by Sigma Aldrich (St. Louis, MO, USA). Electrospray ionization-mass spectrometry, on a micromass-platform LC instrument by Waters Micromass Technologies (Milford, MA, USA) was used to identify the products. Indium chloride (^111^InCl_3_) was purchased from Biomedica Life Sciences SA (Athens, Greece). Radiochemical HPLC analyses were performed on a Waters chromatograph (Waters, Vienna, Austria) with a 600E multi-solvent delivery system coupled to twin detection instrumentation comprising a Waters 2998 photodiode array UV detector and a Gabi γ-detector (Raytest, RSM Analytische Instrumente GmbH, Germany). Data processing and chromatographic control were conducted using the Empower software. Analyses were performed on an XTerra RP-18 (5 μm, 4.6 × 150 mm) cartridge column (Waters, Germany) and on a Symmetry Shield RP-18 (5 μm, 3.9 × 20 mm) column (Waters, Germany). Radioactivity measurements were conducted in an automated well-type γ-counter (NaI(Tl) crystal, Canberra Packard Auto-Gamma 5000 series model, Schwadorf, Austria) calibrated for ^111^In.

#### *Synthesis of conjugates*

SPPS was performed using the standard 9-fluorenyl-methoxycarbonyl (Fmoc)/tert-butyl (^t^Bu) methodology. The AT1S and AT2S amino acid sequences (DOTA-Ala^1^-Gly^2^-Cys^3^-Lys^4^-Asn^5^-Phe^6^-Phe^7^-Trp^8^-Lys^9^-Thr^10^-Phe^11^-Thr^12^-Ser^13^-Cys^14^-OH and DOTA-Ala^1^-Gly^2^-Cys^3^-Lys^4^-Asn^5^-Phe^6^-Phe^7^-DTrp^8^-Lys^9^-Thr^10^-Phe^11^-Thr^12^-Ser^13^-Cys^14^-OH, respectively) were assembled on H-L-Cys(Trt)-2-Chlorotrityl resin (substitution 0.55 mmol/g). Coupling of each amino acid was performed with a threefold molar excess of Fmoc-amino acid, using 1-hydroxybenzotriazol (HOBt) (4.5-fold molar excess) and N,N′-diisopropylcarbodiimide (DIC) (3.3-fold molar excess) as activating agents, in dimethylformamide (DMF). After a period of 2.5 to 3 h, the completeness of the reaction was monitored by the standard ninhydrin test. In case of incomplete coupling, the coupling procedure was repeated prior to N^α^-Fmoc protecting group removal. Fmoc deprotection was performed by the addition of 25% piperidine in DMF for 15 to 25 min. Finally, DOTA-tris(^t^Bu)ester (threefold molar excess) was coupled at the N-terminus of the amino acid chain using HOBt (4.5-fold molar excess) and DIC (3.3-fold molar excess) in DMF. Removal of lateral chain protecting groups and cleavage from the resin was achieved by 4-h incubation in a cleavage cocktail comprising TFA:triethylaminosilane (TES):1,2-ethanedithiol:anisole:H_2_O 93:3:2:1:1 *v*/*v*/*v*/*v*/*v*. The cleavage mixture was evaporated, and the free peptide conjugates were precipitated with diethyl ether and filtered. The crude peptides were dissolved in AcOH-H_2_O 4:1 to a final concentration of 2 mg/mL, and iodine (10-fold molar excess) was added in one portion. The reaction mixture was left to react for 15 to 25 min at 25 °C. After the oxidation was complete, as indicated by the Ellmann test and HPLC monitoring, the reaction was quenched by diluting to twice the volume with H_2_O, and the iodine was extracted in CCl_4_. The aqueous phase was lyophilized, and the products were purified by semi-preparative HPLC on an RP-C18 support using a linear gradient from 20% to 60% MeCN (+0.1% TFA, *v*/*v*) for 40 min at a 2-mL/min flow rate. Eluted peptides were lyophilized immediately. Finally, HPLC analysis was used to monitor the purity of peptides applying two different systems: (a) HPLC system Mod.10 ÄKTA from Amersham Biosciences (Piscataway, NJ, USA) with an Alltech Nucleosil C18 column (5 μm, 4.6 × 250 mm) eluted at a flow rate of 1 mL/min with a linear gradient of 0% B to 40% B in 40 min, with A = 0.1% TFA (*v*/*v*) and B = MeCN containing 0.1% TFA (*v*/*v*) - system A; and (b) Waters chromatograph with 600E multi-solvent delivery system coupled to a Waters 2998 photodiode array UV detector using an RP-18 XTerra (5 μm, 4.6 × 150 mm) cartridge column eluted at a flow rate of 1 mL/min with a linear gradient of 0% B to 60% B in 60 min, with A = 0.1% TFA (*v*/*v*) and B = pure MeCN - system B. Electrospray mass spectrometry (ES-MS) was conducted in order to confirm formation of the desired products.

#### *Radiolabeling with*^*111*^*In*

For ^111^In labeling, ^111^InCl_3_ in 50 mM HCl at a 370- to 740-MBq/mL activity concentration was used. Labeling was conducted by adding 10 nmol AT1S/AT2S analog per 37 to 74 MBq of ^111^InCl_3_ in 0.1 M sodium acetate buffer and 10 mM sodium ascorbate. Typical end pH was 4.6. Labeling was completed after incubation in a boiling water bath for 20 min [13]. Prior to HPLC quality control EDTA in 0.1 M acetate buffer was added to a final concentration of 1 mM to the labeling reaction mixture as a ‘free’ ^111^In^3+^ scavenger.

### Biology

#### *Reagents*

All reagents were of best grade available and were purchased from common suppliers. The sst_2_-specific antibody R2-88 was from Agnes Schönbrunn (Houston, TX, USA), and the sst_3_-specific antibody (SS-850) was purchased from Gramsch Laboratories, Schwabhausen, Germany. The secondary antibody, Alexa Fluor 488 goat anti-rabbit IgG (H + L), was from Molecular Probes, Inc. (Eugene, OR, USA). SS14 was provided by Prof. JE Rivier (The Salk Institute, La Jolla, CA, USA).

#### *Cell lines and animal experiments*

The HEK293 cell line expressing the human T7-epitope-tagged sst_2_ receptor (HEK-sst_2_) or the human sst_3_ or sst_5_ receptor (HEK-sst_3_, HEK-sst_5_) were kindly provided by S. Schultz (Institute of Pharmacology and Toxicology, University Hospital, Friedrich Schiller University Jena, Germany) and cultured as previously described [38,39]. The rat pancreatic tumor cell line AR4-2J endogenously expressing sst_2_ was kindly provided by Prof. S. Mather (St. Bartholomew’s Hospital, London, UK) and cultured as previously described [37]. All culture reagents were from Gibco BRL, Life Technologies (Grand Island, NY, USA) or from Biochrom KG Seromed (Berlin, Germany). Animal experiments were carried out in compliance with European and national regulations and were approved by national authorities. For biodistribution experiments, in-house male Swiss albino mice (30 ± 5 g) were used. For experimental tumor models, in-house SCID mice of 7 weeks of age were used, and the animals were kept under aseptic conditions until biodistribution was performed.

#### *Receptor autoradiography for hsst*_*1-5*_

Cell membrane pellets were prepared from human sst_1_-expressing CHO cells, sst_2_-, sst_3_-, sst_4_-expressing CCL39 cells, and sst_5_-expressing HEK293 cells and stored at −80 °C. Receptor autoradiography was performed on 20-μm-thick cryostat (Microm HM 500, Walldorf, Germany) sections of the membrane pellets, mounted on microscope slides, and then stored at −20 °C as previously described [39,40]. For each of the tested compounds, complete displacement experiments with the universal SS28 radioligand [Leu^8^,DTrp^22^^125^I-Tyr^25^SS28 (^125^I-[LTT]SS28) (74 GBq/mmol; Anawa, Wangen, Switzerland) using 15,000 cpm/100 μL and increasing concentrations of the unlabeled peptide ranging from 0.1 to 1,000 nM were performed. As control, unlabeled SS28 was run in parallel using the same increasing concentrations. The sections were incubated with ^125^I-[LTT]SS28 for 2 h at room temperature in 170 mmol/L Tris–HCl buffer (pH 8.2), containing 1% BSA, 40 mg/L bacitracin, and 10 mmol/L MgCl_2_ to inhibit endogenous proteases. The incubated sections were washed twice for 5 min in cold 170 mmol/L Tris–HCl (pH 8.2) containing 0.25% BSA. After a brief dip in 170 mmol/L Tris–HCl (pH 8.2), the sections were dried quickly and exposed for 1 week to Kodak BioMax MR film (Rochester, NY, USA). IC_50_ values were calculated after quantification of the data using a computer-assisted image processing system as described previously [39]. Tissue standards (Autoradiographic ^125^I] and/or ^14^ C] microscales, GE Healthcare; Little Chalfont, UK) that contain known amounts of isotope, cross-calibrated to tissue-equivalent ligand concentrations were used for quantification [39-42].

#### *Sst*_*2*_*-and sst*_*3*_*-internalization assay*

Immunofluorescence microscopy-based internalization assay for sst_2_ and sst_3_ was performed as previously described [38,43]. HEK-sst_2_ and HEK-sst_3_ cells were grown on poly-dLys (20 μg/mL) (Sigma-Aldrich, St. Louis, MO, USA) coated 35-mm four-well plates (Cellstar, Greiner Bio-One GmbH, Frickenhausen, Germany). Cells were treated for 30 min at 37 °C in growth medium with increasing concentrations ranging between 1 and 100 nM of either AT2S or SS14 (positive control). The cells were then rinsed twice with PS (100 mM phosphate buffer containing 0.15 M sucrose), fixed and permeabilized for 7 min with cold methanol (−20 °C), rinsed twice with PS, and then blocked for 60 min at room temperature with PS containing 0.1% BSA. Subsequently, the cells were incubated for 60 min at room temperature with the sst_2_ specific primary antibody R2-88 or the sst_3_ specific primary antibody SS-850, both diluted 1:1,000 in PS and then washed 3 × 5 min with PS containing 0.1% BSA. The cells were then incubated for 60 min at room temperature in the dark with the secondary antibody Alexa Fluor 488 goat anti-rabbit IgG (H + L) diluted in PS (1:600), subsequently washed 3 × 5 min with PS containing 0.1% BSA, and embedded with PS/glycerol 1:1 and covered with a glass cover slip. The cells were imaged using a Leica DM RB immunofluorescence microscope (Leica, Deerfield, IL, USA) and an Olympus DP10 camera (Olympus Corporation, Shinjuku, Tokyo, Japan).

#### *Radioligand internalization assay*

For radioligand internalization experiments, the rsst_2_-positive cell line AR4-2J was used. Cells were grown to confluence for 48 h in six-well plates. On the day of the experiment, cells were washed twice with ice-cold internalization medium prepared with F-12-K nutrient mixture supplemented by 1% (*v*/*v*) fetal bovine serum. The cells were supplied with fresh medium (1.2 mL), and approximately 300,000 cpm/150 μL [^111^In]AT1S/[^111^In]AT2S (corresponding roughly to 2 pmol total peptide) was added to the medium followed by 0.5% BSA PBS alone (150 μL, total series) or by a 1-μM Tate solution in 0.5% BSA PBS (150 μL, nonspecific series). Cells were incubated at 37 °C in triplicates for each time point of 5, 15, 30, 60, and 120 min. Incubation was interrupted by removal of the medium and rapid rinsing with ice-cold 0.5% BSA PBS. Cells were then incubated twice for 5 min at ambient temperature in acid wash buffer (50 mM glycine buffer with pH 2.8, 0.1 M NaCl). The supernatant was collected (membrane-bound radioligand fraction) each time and pooled, and the cells were rinsed with 0.5% BSA PBS. Cells were lysed by treatment in 1 N NaOH, and cell radioactivity was collected (internalized radioligand fraction). Considering that total activity comprises membrane-bound plus internalized activity, the percent internalized activity versus the selected 5-, 15-, 30-, 60-, and 120-min time intervals could be calculated applying the Microsoft Excel program.

#### *Metabolism in blood*

A 150-μL bolus containing [^111^In]AT1S/[^111^In]AT2S (11 to 22 MBq, 3 nmol total peptide) was injected in the tail vein of healthy male Swiss albino mice. The animals were kept for 5 min in cages with free access to water. They were sacrificed by cardiac puncture under ether anesthesia, and blood was withdrawn with a syringe and immediately placed in a pre-chilled EDTA-containing polypropylene vial on ice. Blood samples were centrifuged at 2,000 × *g* at 4 °C for 10 min. The supernatant (>90% radioactivity recovered) was collected, and an equal volume of MeCN was added. The mixture was centrifuged for 10 min at 15,000 × *g* at 4 °C. The supernatant (>90% recovery of radioactivity) was collected, and the organic solvent was removed under N_2_-flux; the residue was redissolved in physiological saline, passed through a 0.22-μm Millex-GV filter (Millipore, Milford, USA) (>90% recovery of radioactivity), and analyzed by RP-HPLC (>96% radioactivity recovered). For establishing the retention times (*t*_R_ in min) of parent radiopeptides, blood samples were co-injected with the respective [^111^In]AT1S or [^111^In]AT2S and analyzed by RP-HPLC applying the same conditions.

#### *In vivo distribution experiments in healthy mice*

For tissue distribution experiments, male Swiss albino mice were each injected with a 100-μL bolus containing [^111^In]AT1S or [^111^In]AT2S (37 to 74 kBq, 10 pmol of total peptide) via the tail vein. In the *in vivo* receptor blockade animal group, excess Tate (50 nmol) was administered intravenously (iv) together with the radioligand. Animals were sacrificed in groups of four at 4- and 24-h time points post injection (pi). Blood and urine were immediately collected, and the organs of interest were excised and weighed; their radioactivity content was measured in an automatic gamma counter using proper standards of the injected dose. Tissue distribution data were calculated as percent injected dose per gram (%ID/g) applying a suitable algorithm.

#### *In vivo distribution experiments in AR4-2J tumor-bearing mice*

In the flanks of each of female SCID mice, inocula (150 μL) containing a suspension of 0.8 × 10^7^ AR4-2J cells in PBS buffer were subcutaneously injected. Tumors of substantial size were grown within 12 days, whereupon biodistribution experiments were performed selectively for [^111^In]AT2S. Animals were injected in the tail vein with a 100-μL bolus containing [^111^In]AT2S (37 to 74 kBq, 10 pmol of total peptide); three animals were co-injected with excess Tate (50 nmol) together with the radioligand (blocked animals). Mice were sacrificed at 4 h pi, and biodistribution was studied as described above.

#### *In vivo distribution experiments in HEK293-hsst*_*2A*_^*+*^*, -hsst*_*3*_^*+*^*or -hsst*_*5*_^*+*^*tumor-bearing mice*

In the flanks of SCID mice, inocula (150 μL) containing a suspension of 1.8 × 10^7^ HEK293-hsst_2A_^+^, -hsst_3_^+^ or -hsst_5_^+^ cells in PBS were subcutaneously injected. Tumors of substantial size were grown within 3 weeks, whereupon biodistribution experiments were performed. Animals were injected in the tail vein with a 100-μL bolus containing [^111^In]AT2S (37 to 74 kBq, 10 pmol of total peptide) and were sacrificed at 4 h pi; for *in vivo* blockade, mice were co-injected with either excess DP2 (35 nmol; HEK293-hsst_2A_^+^ tumors) or with excess AT2S (35 nmol; HEK293-hsst_3_^+^ and -hsst_5_^+^ tumors), and biodistribution was conducted as described above.

#### *Statistical analysis*

The *in vivo* data presented as mean %ID/g ± SD (*n* ≥ 4) were statistically analyzed with Student’s *t* test (Prism^TM^ 2.01, GraphPad Software, San Diego, CA, USA). Analyses were 2-tailed, and a *P* value < 0.05 was considered statistically significant.

## Results and discussion

### Results

#### *Synthesis of conjugates*

**Figure 1:**
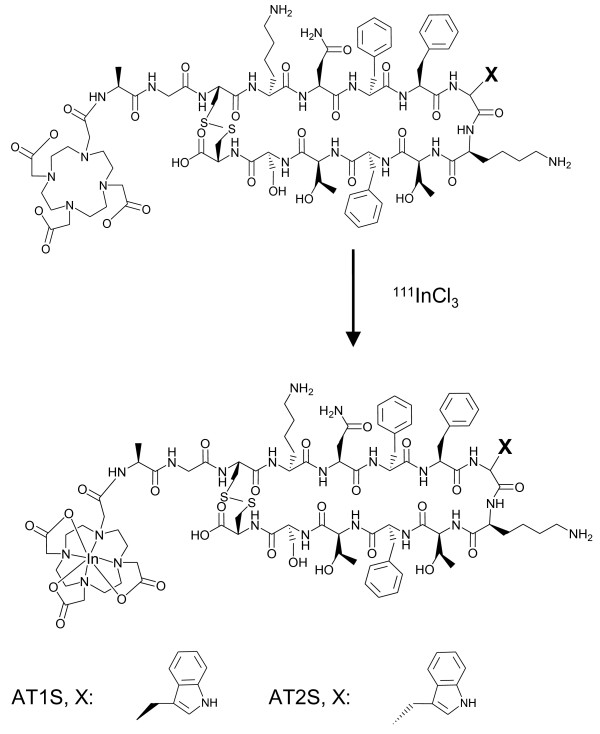
**Molecular structure of AT1S/AT2S and their**^**111**^**In analogs.**

The linear amino acid AT1S and AT2S sequences were assembled on the solid support applying the Fmoc/^t^Bu methodology, and the DOTA-protected chelator was coupled at the N-terminus. The DOTA-peptide conjugates were cleaved from the solid support, and the lateral protecting groups were removed by TFA treatment. Cyclization was conducted with iodine oxidation in solution and was monitored by analytical HPLC. The cyclized products (Figure 1) were isolated by semi-preparative HPLC and lyophilized. Product purity was assessed by analytical HPLC; ES-MS data were consistent with the expected formula (Table 1).

**Table 1:** Analytical data for AT1S and AT2S

**Analog**	**Purity**	**Expected MW (*m/z*)**	**ES-MS**	**System A**^**a**^***t***_**R**_**(min)**	**System B**^**b**^***t***_**R**_**(min)**
AT1S	98%	2024.29	-	36.9	41.9
		[M + H^+^] = 2025.29			
		[M + 2 H^+^] / 2 = 1013.14	-		
		[M + 3 H^+^] / 3 = 675.76	675.60 (100)^*^		
AT2S	90%	2024.29		35.8	39.3
		[M + H^+^] = 2025.29			
		[M + 2 H^+^] / 2 = 1013.14	1012.66 (60)^*^		
		[M + 3 H^+^] / 3 = 675.76	675.72 (40)^*^		

^a^System A and ^b^system B are described in the ‘Methods’ section. ^*^Numbers in parenthesis signify relative intensities of peaks in the MS-spectra. MW, molecular weight.

#### *Radiolabeling*

**Figure 2:**
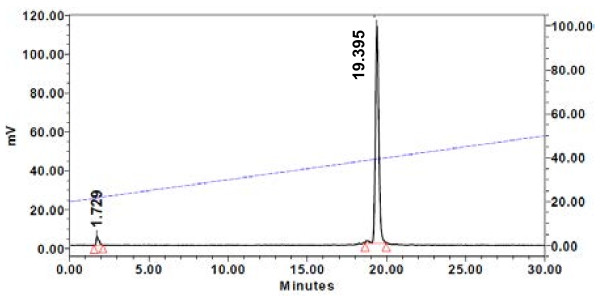
**HPLC analysis of [**^**111**^**In]AT2S.** Representative radiochromatogram of [^111^In]AT2S labeling reaction mixture performed on a Waters 600 HPLC. A Symmetry Shield RP18 column was eluted at a flow rate of 1 mL/min with a linear gradient of 20% *B* to 50% *B* in 30 min; *A* = 0.1% TFA (*v*/*v*), and *B* = MeCN.

Labeling of ATIS and AT2S with ^111^In (Figure 1) was achieved by a 20-min incubation of the analogs in acidic medium at 90 °C in the presence of ^111^InCl_3_, according to published protocols [13,44]. A >96% radiometal incorporation was typically shown by HPLC analysis on an RP column; a representative radiochromatogram of ^111^In]AT2S quality control is shown in Figure 2.

#### *Determination of hsst*_*1-5*_*profile*

The IC_50_ values of ATIS and AT2S for all five somatostatin receptor subtypes are summarized in Table 2. Data were acquired by receptor autoradiography assays in cells selectively expressing one of the five hsst_1-5_; [Leu^8^,DTrp^22^,^125^I-Tyr^25^]SS28 was used as pansomatostatin radioligand and SS14 and SS28 as controls. Both analogs, AT1S and AT2S, exhibit a clear pansomatostatin profile with a high affinity binding to all five hsst_1-5_. However, AT1S and AT2S show a slightly lower affinity for sst_1_ and sst_5_ compared with the natural somatostatins, SS14 and SS28.

**Table 2:** **Affinity profile (IC**_**50**_**in nanomolar) of AT1S and AT2S for the human sst**_**1-5**_**receptors**

**Code**	**hsst**_**1**_	**hsst**_**2**_	**hsst**_**3**_	**hsst**_**4**_	**hsst**_**5**_
SS14	1.9 ± 0.5 (5)	0.7 ± 0.2 (5)	3.3 ± 1.7 (4)	1.6 ± 0.8 (4)	4.2 ± 0.7 (3)
SS28	2.7 ± 0.5 (3)	2.5 ± 0.1 (3)	2.2 ± 0.6 (3)	2.1 ± 0.4 (3)	2.0 ± 0.2 (3)
AT1S	5.1 ± 1.4 (3)	2.8 ± 0.3 (3)	1.8 ± 0.6 (3)	2.5 ± 0.6 (3)	17 ± 3 (3)
AT2S	14 ± 2 (3)	1.5 ± 0.3 (3)	2.4 ± 0.5 (3)	3.7 ± 0.7 (3)	12 ± 2 (3)

Mean ± SEM (numbers in parentheses = number of experiments). The profile for the human sst_1-5_ receptors was determined by receptor autoradiography experiments; [Leu^8^,DTrp^22^,^125^I-Tyr^25^]SS28 was used as the radioligand and SS14 and SS28 as control compounds.

#### *Sst*_*2*_*- and sst*_*3*_*-internalization assay*

**Figure 3:**
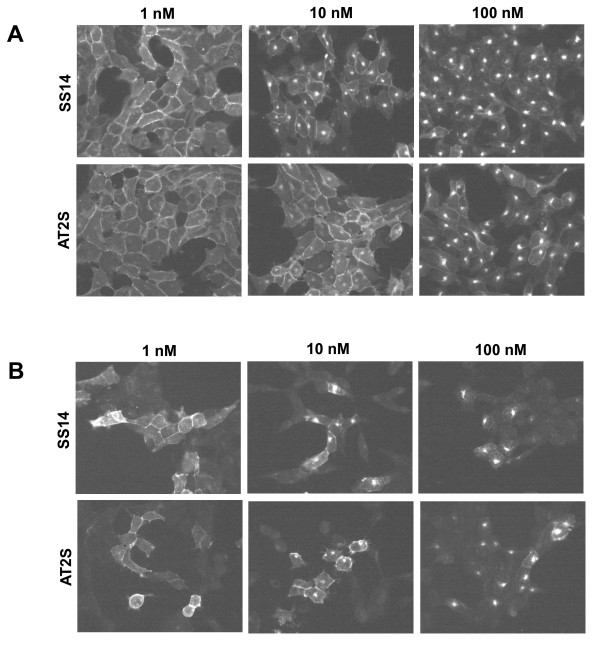
**Sst**_**2 **_**or sst**_**3 **_**receptor internalization in HEK-sst**_**2 **_**or HEK-sst**_**3 **_**determined by immunofluorescence microscopy.** HEK-sst_2_**(A)** or HEK-sst_3_**(B)** cells were treated for 30 min at 37 °C with increasing concentrations ranging between 1 and 100 nM of either AT2S or SS14 (positive control) and then processed for immunofluorescence microscopy. AT2S is an agonist at sst_2_ and sst_3_ since it stimulates internalization of both receptors.

The ability of AT2S to stimulate sst_2_ or sst_3_ internalization in HEK-sst_2_ and HEK-sst_3_ cells was analyzed using an immunofluorescent-based internalization assay. Figure 3 illustrates that AT2S exhibits similar agonistic properties as the natural SS14 for sst_2_ (Figure 3A) and for sst_3_ (Figure 3B) in respect of stimulating receptor internalization.

#### *Internalization of radioligands*

**Figure 4:**
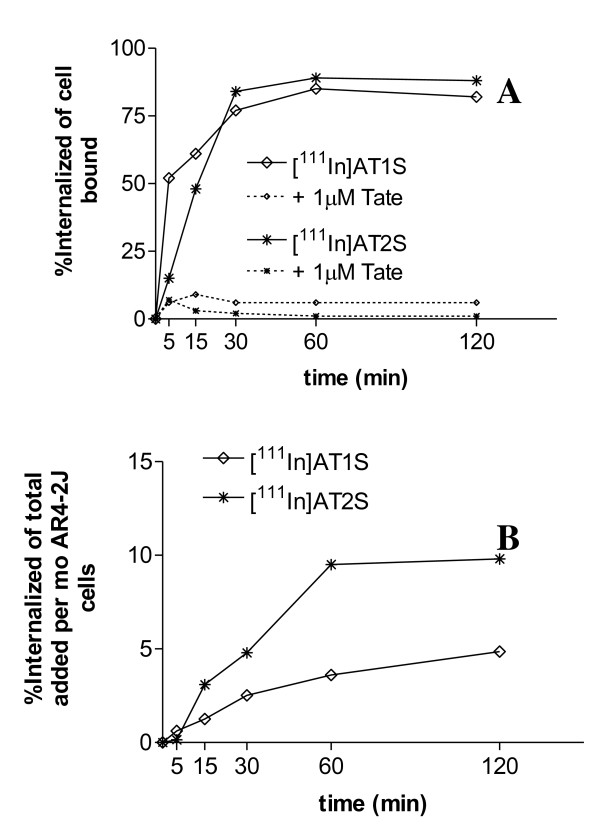
**Comparative internalization of [**^**111**^**In]AT1S and [**^**111**^**In]AT2S in AR4-2J cells.(A)** Internalized fraction calculated versus cell-associated activity and **(B)** internalized fraction calculated per million cells versus total added radioactivity; nonspecific internalization, as determined in the presence of 1 μM Tate in the medium, is indicated by dashed lines.

The internalization properties of the radiopeptides [^111^In]AT1S and [^111^In]AT2S were studied in rsst_2A_^+^ AR4-2J cells at 37 °C with or without excess Tate. The internalization of both analogs was rapid and rsst_2A_-mediated, as shown by the significant decrease of internalization levels manifested in the presence of excess Tate (Figure 4A,B). More than 75% of cell-associated activity internalized within 30 min remaining at this level up to 2 h for both radiopeptides (Figure 4A). At 120 min, 5% and 10% of the total added radioactivity internalized for [^111^In]AT1S and [^111^In]AT2S, respectively, revealing a significantly more efficient internalization process for the DTrp^8^ analog (Figure 4B).

#### *Metabolism in blood*

**Figure 5:**
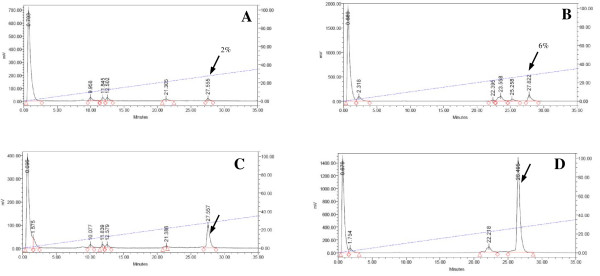
**Comparative* in vivo *stability of [**^**111**^**In]AT1S and [**^**111**^**In]AT2S.** Analysis by HPLC of murine blood collected 5 min after injection of **(A)** [^111^In]AT1S and **(B)** [^111^In]AT2S. Co-injection with **(C)** [^111^In]AT1S and **(D)** [^111^In]AT2S radioligands show the *t*_R_ of the respective intact radiopeptides. Analyses were performed on a Waters 600 HPLC system with a Symmetry Shield RP18 column eluted at a flow rate of 1 mL/min with a linear gradient of 0% B to 50% B in 50 min, with A = 0.1% TFA (*v*/*v*) and B = MeCN; the position of original peptide elution is indicated by the arrow, and the number by the arrow shows the percentage (%) of intact peptide detected in the blood.

Both [^111^In]AT1S and [^111^In]AT2S showed suboptimal stability in the blood stream of healthy mice. As evidenced by HPLC analysis of murine blood collected 5 min after injection of the radioligands, both analogs degraded to at least one major radiometabolite eluting with the solvent front. Traces of additional metabolites having different elution patterns for the two analogs were also observed (Figure 5A,B). The DTrp^8^ containing radiopeptide showed higher metabolic stability with 6.5% of intact [^111^In]AT2S still detected in this period versus only 2% of [^111^In]AT1S found intact under the same conditions. The *t*_R_ of the intact analogs was established after co-injection with radiolabeled samples not administered in mice (Figure 5 C,D, respectively).

#### *Biodistribution in healthy mice*

**Figure 6:**
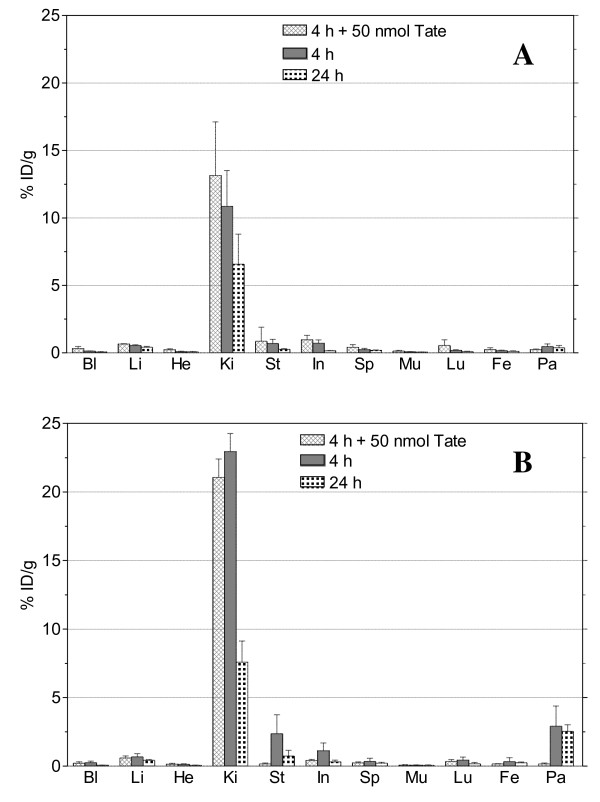
**Biodistribution data of (A) [**^**111**^**In]AT1S and (B) [**^**111**^**In]AT2S.** Data are acquired at 4 and 24 h after iv injection of [^111^In]AT1S and [^111^In]AT2S in healthy male Swiss albino mice. Data are given as mean of %ID/g ± SD, *n* ≥ 4 per time point; for the *in vivo* sst_2_ blockade group at 4 h pi, animals received 50 nmol Tate along with the radioligand. Bl, blood; Li, liver; He, heart; Ki, kidneys; St, stomach; In, intestines; Sp, spleen; Mu, muscle; Lu, lung; Fe, femur; and Pa, pancreas.

Tissue distribution data of [^111^In]AT1S and [^111^In]AT2S in healthy male Swiss albino mice for the 4- and 24-h time points are summarized as %ID/g in Figure 6A,B, respectively. Both analogs showed a rapid clearance from blood and background tissues with minimal residual activity in pool organs. [^111^In]AT1S failed to show specific uptake in the sst_2_-rich pancreas (<0.5%ID/g at 4 h pi) in contrast with [^111^In]AT2S, which clearly showed receptor-specific uptake in this organ (2.9%ID/g at 4 h pi versus 0.17%ID/g at 4 h pi + excess Tate) as well as in the stomach and intestines. On the other hand, kidney uptake was unfavorably higher for [^111^In]AT2S (22.9%ID/g versus 10.9%ID/g for [^111^In]AT1S at 4 h pi). However, renal values declined over time for both analogs, reaching comparable levels at 24 h pi (6.6%ID/g for [^111^In]AT1S and 7.6%ID/g for [^111^In]AT2S).

#### *Biodistribution in AR4-2J tumor-bearing mice*

Results of [^111^In]AT2S tissue distribution in SCID mice bearing AR4-2J experimental tumors are included in Table 3, as %ID/g at 1 and 4 h pi. Biodistribution data in SCID mice were consistent to the findings of healthy mice tissue distribution with specific uptake shown in the pancreas (5.13%ID/g versus 0.15%ID/g + excess Tate at 4 h pi) and the gastrointestinal tract. Uptake in the rsst_2A_^+^ tumor was shown to be specific as well, as suggested by the significantly reduced tumor values found during co-injection of excess Tate (1.82%ID/g at 4 h versus 0.21%ID/g + excess Tate at 4 h pi).

**Table 3:** **Cumulative biodistribution data of [**^**111**^**In]AT2S in AR4-2J and HEK293-hsst**_**2A**_^**+**^**, -hsst**_**3**_^**+**^**, and -hsst**_**5**_^**+**^**tumor-bearing SCID mice**

**[**^**111**^**In]AT2S (%ID/g tissue ± SD)**									
**Organs**	**AR4-2J**			**HEK293-hsst**_**2A**_^**+**^		**HEK293-hsst**_**3**_^**+**^		**HEK293-hsst**_**5**_^**+**^	
	**1 h**	**4 h**	**4 h + Tate**^**a**^	**4 h**	**4 h + DP2**^**b**^	**4 h**	**4 h + AT2S**^**c**^	**4 h**	**4 h + AT2S**^**c**^
Blood	1.34 ± 0.21	0.19 ± 0.02	0.26 ± 0.03	0.23 ± 0.07	0.66 ± 0.35	0.19 ± 0.03	0.2 ± 0.10	0.1 ± 0.04	0.23 ± 0.03
Liver	1.27 ± 0.09	0.95 ± 0.21	1.02 ± 0.11	1.31 ± 0.36	0.963 ± 0.67	1.02 ± 0.19	1.09 ± 0.2	1.1 ± 0.08	1.25 ± 0.09
Heart	0.67 ± 0.12	0.18 ± 0.05	0.17 ± 0.04	0.22 ± 0.05	0.38 ± 0.10	0.15 ± 0.04	0.15 ± 0.01	0.15 ± 0.02	0.18 ± 0.03
Kidneys	23.83 ± 4.28	21.10 ± 4.43	24.41 ± 9.15	23.20 ± 7.04	25.51 ± 3.70	23.42 ± 4.69	23.55 ± 8.96	24.12 ± 1.21	24.68 ± 2.20
Stomach	3.23 ± 0.77	1.81 ± 0.47	0.18 ± 0.07***	1.96 ± 0.53	1.29 ± 0.36	3.41 ± 0.97	0.19 ± 0.05***	3.06 ± 0.73	0.17 ± 0.2***
Intestines	1.98 ± 0.29	1.92 ± 0.25	0.77 ± 0.58*	2.00 ± 0.48	1.74 ± 1.02	2.39 ± 0.43	0.54 ± 0.2***	1.54 ± 0.09	0.57 ± 0.20***
Spleen	0.84 ± 0.21	1.10 ± 0.63	0.61 ± 0.18	0.46 ± 0.11	0.50 ± 0.36	0.82 ± 0.11	0.98 ± 0.23	0.78 ± 0.07	1.08 ± 0.16
Muscle	0.22 ± 0.02	0.06 ± 0.01	0.07 ± 0.01	0.07 ± 0.02	0.07 ± 0.03	0.06 ± 0.01	0.07 ± 0.02	0.06 ± 0.01	0.06 ± 0.02
Lung	2.05 ± 0.52	1.43 ± 1.36	3.30 ± 4.93	1.37 ± 0.55	0.47 ± 0.08	0.68 ± 0.29	0.73 ± 0.14	0.56 ± 0.28	0.83 ± 0.13
Femur	0.49 ± 0.12	0.40 ± 0.24	0.19 ± 0.02	0.24 ± 0.09	0.21 ± 0.11	0.23 ± 0.03	0.23 ± 0.04	-	-
Pancreas	7.35 ± 0.74	5.13 ± 0.75	0.15 ± 0.05**	5.73 ± 1.28	0.16 ± 0.10***	3.33 ± 0.4	0.11 ± 0.01***	2.79 ± 0.4	0.43 ± 0.54***
Tumor	3.05 ± 0.51	1.82 ± 0.36	0.21 ± 0.17***	1.49 ± 0.2	0.27 ± 0.20***	1.24 ± 0.27	0.32 ± 0.06***	0.41 ± 0.12	0.22 ± 0.006*

*Significant (*p* < 0.05), and ** and ***highly significant (*p* < 0.005) difference between blocked and unblocked animals (Student’s *t* test). ^a^Co-injection of 50 nmol Tate; ^b^co-injection of 35 nmol DP2; ^c^co-injection of 35 nmol AT2S.

#### *Biodistribution in HEK293-hsst*_*2A*_^*+*^*, -hsst*_*3*_^*+*^*or -hsst*_*5*_^*+*^*tumor-bearing mice*

In SCID mice bearing HEK293-hsst_2A_^+^, -hsst_3_^+^ or -hsst_5_^+^ tumors, [^111^In]AT2S showed similar biodistribution in all organs, as indicated in Table 3. High and specific uptake was observed in both hsst_2A_^+^ (1.49%ID/g versus 0.27%ID/g + excess DP2 at 4 h pi) and -hsst_3_^+^ tumors (1.24%ID/g versus 0.32%ID/g + excess AT2S at 4 h pi). In the HEK293-hsst_5_^+^ tumors, the uptake was lower (0.41%ID/g versus 0.22%ID/g + excess AT2S at 4 h pi), a finding consistent with the lower affinity of AT2S for the hsst_5_^+^ (12 ± 2 nM) found *in vitro* (Table 2).

## Discussion

The success of OctreoScan® and related cyclic octapeptide sst_2_-seeking radioligands in the diagnosis and treatment of certain human tumors relies both on their high metabolic stability and on the prevalence and high density of sst_2_ expression in these tumors [11-19]. Soon, it became apparent that sst_2_-mediated internalization of radioligands into cancer cells represents a key element for the success of this strategy. Intracellular accumulation of the radiolabel has translated into higher contrast images and to better tumoricidal responses.

On the other hand, recent studies have reported not only on the concomitant expression of at least one alternative sst_1-5_ subtype in tumors already expressing the sst_2_, but also in tumors devoid of sst_2_ expression [6,7,15,20-26]. This finding provides the opportunity to use radiolabeled somatostatin analogs with an extended sst_1-5_ affinity profile, which will consequently interact with more binding sites on the tumor than those limited to sst_2_. In this way, the diagnostic and therapeutic indications will be broadened to include more tumor types, while diagnostic sensitivity and therapeutic efficacy will improve. Such ‘pansomatostatin-like’ radioligands should possess sufficient metabolic stability to be able to reach their target after entry into the bloodstream. At the same time, their capacity to internalize in sst_2_^+^-cancer cells should not be compromised in order to promote accumulation in most sst_1-5_^+^-human tumors whereby sst_2_ expression is dominant [27-30]. It is interesting to note that pansomatostatin-like radioligands failing to internalize after binding to sst_2_*in vivo* indeed showed poor uptake in sst_2_^+^ tissues in mouse models [31,32]. On the other hand, multi-sst affine and well sst_2_-internalizing radioligands, such as radiolabeled DOTA-NOC [33], are expected to miss sst_1_-expressing tumors in patients [23-26].

The above requirements prompted us to consider the use of native SS14 for radioligand development. It is interesting to note that a SS14-derived radiopeptide, ^111^In-[DTPA,DAla^1^,DTrp^8^,Tyr^11^SS14, was previously studied in healthy mice and compared to OctreoScan® [34]. This analog displayed a pansomatostatin-like profile and showed equivalent to OctreoScan® levels of specific uptake in key target organs, such as the pituitary, the pancreas, and the adrenals, implying that SS14-based radioligands do have opportunities of good sst-targeting *in vivo*, including the sst_2_. No other information on similar SS14-based radiopeptides is available.

Therefore, we have decided to couple DOTA to the N-terminus of native and non-modified SS14. In this way, AT1S was first generated with the purpose to serve as a lead compound to future structurally modified pansomatostatin-like radiopeptides and as a landmark for their biological evaluation. The universal chelator DOTA has been selected over DTPA with the aim to broaden labeling options beyond ^111^In to numerous other medically attractive bi- and trivalent radiometals. Coupling of DOTA on the Ala^1^ primary amine of SS14 inadvertently leads to N-terminal capping of the peptide chain as well, a strategy often pursued to increase metabolic stability of peptides. In the second analog, AT2S, Trp^8^ was further substituted by DTrp^8^ in our AT1S motif to convey additional metabolic stability. This modification is reported to also facilitate the β-turn conformation of several cyclic somatostatin analogs leading to enriched affinity for the sst_2_[35,36].

Both AT1S and AT2S exhibited a pansomatostatin-like *in vitro* profile, binding to all five sst_1-5_ with affinities in the lower nanomolar range. The presence of DOTA at the N-terminus has caused minor affinity losses for all subtypes, which were more pronounced for sst_1_ and sst_5_. A similar trend was also observed for [DTPA,DAla^1^,DTrp^8^,Tyr^11^]SS14. Of particular interest is the ability of AT2S to induce sst_2_ and sst_3_ internalization *in vitro*, as evidenced by immunofluorescence microscopy. This agonistic behavior for both, sst_2_ and sst_3_, subtypes is similar to native SS14 as it is elicited at comparable concentration levels (≈10 nM). In agreement to this finding, [^111^In]AT1S and [^111^In]AT2S internalized in AR4-2J cells by a sst_2_-mediated process. Within 30 min at 37 °C, ≈80% of cell bound activity was found within the cells. It is interesting to note that [^111^In]AT2S showed faster internalization of total-added radioactivity as compared with [^111^In]AT1S. This difference in internalization rates is reflected in dissimilar uptake of the two radioligands in sst_2_^+^ organs after injection in mice (*vide infra*).

The metabolic fate of [^111^In]AT1S and [^111^In]AT2S was followed 5 min after entry in the bloodstream of mice and revealed their susceptibility to enzymatic degradation. [^111^In]AT1S was almost totally degraded within this period, despite the N-terminal capping conveyed by the ^111^In-DOTA moiety, as compared with native SS14. By Trp^8^/DTrp^8^ substitution in [^111^In]AT2S, the percentage of integer radiopeptide increased threefold while the pattern of detected metabolites changed. These differences, albeit small, may have a significant impact on biodistribution in the case where blood clearance and target delivery rates are fast enough to compensate, at least in part, rapid degradation rates. It is interesting to note that after injection in healthy mice, only [^111^In]AT2S achieves to specifically target sst-binding organs, such as the pancreas, as revealed by co-injection of excess Tate. Pancreatic values remained unchanged from 1 to 24 h pi, whereas renal values substantially declined during this period. In contrast, [^111^In]AT1S failed to show any measurable specific uptake, most probably as a result of its slower sst_2_-mediated internalization combined with its poorer *in vivo* stability. Accordingly, further evaluation in tumor-bearing mice was focused on [^111^In]AT2S.

In mice bearing AR4-2J tumors spontaneously expressing the rat sst_2_, [^111^In]AT2S showed clear specific uptake both in the experimental tumor and in the gut, including the pancreas, stomach, and intestines, as confirmed by suitable *in vivo* sst_2_ blockade with excess of sst_2_-selective Tate. Similarly high and specific uptake was observed in HEK-hsst_2A_^+^ and HEK-sst_3_^+^ tumors at 4 h pi, although the affinity of AT2S for the hsst_2A_ was slightly higher as for the hsst_3_, and AT2S showed a similar agonistic capacity in triggering the internalization of both subtypes *in vitro*. On the other hand, [^111^In]AT2S showed a much lower, although still specific, uptake in the HEK-hsst_5_^+^ implants. This decrease may be attributed to its ≈ 10-fold lower affinity for hsst_5_. It should be stressed, however, that individual hsst-expression levels on transfected HEK cells may be different, thereby affecting radioligand uptake.

## Conclusions

In summary, native SS14 and its DTrp^8^ analog were functionalized with the universal chelator DOTA to allow for labeling with most interesting diagnostic and therapeutic radiometals. The respective AT1S prototype and its DTrp^8^ derivative, AT2S, were labeled with ^111^In, and several *in vitro* and *in vivo* properties of resulting (radio)ligands were investigated. According to the data obtained, both AT1S and AT2S show a pansomatostatin-like affinity profile, and AT2S displays a clear agonistic character for hsst_2_ and hsst_3_*in vitro*. In contrast with previously reported pansomatostatin-like radioligands showing poor sst_2_-related internalization, [^111^In]AT1S and [^111^In]AT2S do internalize in AR4-2J cells via a sst_2_-mediated mechanism. This parameter is promising for *in vivo* application, and it was more pronounced for [^111^In]AT2S. Furthermore, after injection in mice, [^111^In]AT2S survived longer in circulation to effectively target physiological somatostatin binding sites, such as the pancreas. Likewise, [^111^In]AT2S specifically localized in experimental tumors in SCID mice which selectively expressed one of sst_2_ (both of rat and human origin), hsst_3_, or hsst_5_. To our knowledge, this is the first comprehensive study that systematically explores strengths and weaknesses of employing native SS14-derived radioligands for nuclear oncology applications. It has demonstrated that the AT1S lead structure is promising for radioligand development owing to its pansomatostatin character and its preserved agonistic properties, especially regarding sst_2_ internalization. Furthermore, it has revealed the feasibility of structural modifications to enhance metabolic stability in order to achieve higher tumor uptake. The body of data so far acquired will serve as a landmark in the evaluation of innovative structural interventions on the AT1S lead structure, such as key amino acid replacements and/or changes of ring size, which are currently pursued.

## Abbreviations

DOTA: 1,4,7,10-tetraazacyclododecane-1,4,7,10-tetraacetic acid; DOTA-NOC: [DOTA0,1-Nal3,Thr8]octreotide; DTPA: diethylenetriamine-N,N,N′,N″,N″-pentaacetic acid; hsst: human somatostatin receptor subtype; KE108: Tyr-c[D-diaminobutyric acid-Arg-Phe-Phe-DTrp-Lys-Thr-Phe]; Lanreotide or Somatuline: H-D-2-Nal-c[Cys-Tyr-DTrp-Lys-Val-Cys]-Thr-NH2; Octreotide or Sandostatin: H-DPhe-c[Cys-Phe-DTrp-Lys-Thr-Cys]-Thr(ol); rsst2A: rat somatostatin receptor subtype 2A; SOM230: c[diaminoethylcarbamoyl-HydroxyPro-Phenylglycine-DTrp-Lys-(4-O-benzyl)Tyr-Phe]; somatostatin-14 or SS14: H-Ala-Gly-c[Cys-Lys-Asn-Phe-Phe-Trp-Lys-Thr-Phe-Thr-Ser-Cys]-OH; SPPS: solid phase peptide synthesis; sst: somatostatin receptor subtype; tR: retention time (min) during HPLC analysis.

## Competing interests

The authors declare that they have no competing interests.

## Authors’ contributions

AT was actively engaged in peptide synthesis, radiolabeling, and biological evaluation and assisted in writing the manuscript (ms). TM performed animal studies and drafted most parts of the ms. RC, BW, and JCR were engaged in sst_1-5_ affinity profile determination of AT1/2 S and sst_2_/sst_3_ internalization studies and drafted the corresponding ms sections. PC supervised peptide synthesis and participated in the design of analogs. EPK, MdJ, and JCR edited the ms. BAN designed the overall study and supervised radiochemical work, as well as the generation and final editing of this ms. All authors read and approved the final manuscript.
